# Tinnitus in Normal-Hearing Participants after Exposure to Intense Low-Frequency Sound and in Ménière’s Disease Patients

**DOI:** 10.3389/fneur.2016.00239

**Published:** 2017-01-05

**Authors:** Margarete Anna Ueberfuhr, Lutz Wiegrebe, Eike Krause, Robert Gürkov, Markus Drexl

**Affiliations:** ^1^German Center for Vertigo and Balance Disorders, University Hospital Munich, Ludwig-Maximilians Universität München, Munich, Germany; ^2^Graduate School of Systemic Neurosciences, Ludwig-Maximilians-Universität München, Martinsried, Germany; ^3^Division of Neurobiology, Department Biology II, Ludwig-Maximilians-Universität München, Martinsried, Germany; ^4^Department of Otorhinolaryngology, Head and Neck Surgery, Grosshadern Medical Centre, Ludwig-Maximilians Universität München, Munich, Germany

**Keywords:** tinnitus, low-frequency, Ménière’s disease, bounce phenomenon, endolymphatic hydrops

## Abstract

Tinnitus is one of the three classical symptoms of Ménière’s disease (MD), an inner ear disease that is often accompanied by endolymphatic hydrops. Previous studies indicate that tinnitus in MD patients is dominated by low frequencies, whereas tinnitus in non-hydropic pathologies is typically higher in frequency. Tinnitus of rather low-frequency (LF) quality was also reported to occur for about 90 s in normal-hearing participants after presentation of intense, LF sound (120 dB SPL, 30 Hz, 90 s). LF sound has been demonstrated to also cause temporary endolymphatic hydrops in animal models. Here, we quantify tinnitus in two study groups with chronic (MD patients) and presumably transient endolymphatic hydrops (normal-hearing participants after LF exposure) with a psychophysical procedure. Participants matched their tinnitus either with a pure tone of adjustable frequency and level or with a noise of adjustable spectral shape and level. Sensation levels of matching stimuli were lower for MD patients (mean: 8 dB SL) than for normal-hearing participants (mean: 15 dB SL). Transient tinnitus after LF-exposure occurred in all normal-hearing participants (*N* = 28). About half of the normal-hearing participants matched noise to their tinnitus, the other half chose a pure tone with frequencies below 2 kHz. MD patients matched their tinnitus with either high-frequency pure tones, mainly above 3 kHz, or with a noise. Despite a significant proportion of MD patients matching low-pass (roaring) noises to their tinnitus, the range of matched stimuli was more heterogeneous than previous data suggested. We propose that in those participants with noise-like tinnitus, the percept is probably generated by increased spontaneous activity of auditory nerve fibers with a broad range of characteristic frequencies, due to an impaired ion balance in the cochlea. For tonal tinnitus, additional mechanisms are conceivable: focal hair cell loss can result in decreased auditory nerve firing and a central auditory overcompensation. Also, normal-hearing participants after LF-exposure experience alterations in spontaneous otoacoustic emissions, which may contribute to a transient tonal tinnitus.

## Introduction

Tinnitus is defined as the perception of sound in the absence of external acoustic stimulation and can take various forms from pure tones to more atonal percepts (1, 2).

Tinnitus can be distinguished into two main classes: objective tinnitus and subjective tinnitus. Objective tinnitus is caused by sounds originating from internal sources, e.g., from the patient’s inner ear such as prominent spontaneous otoacoustic emissions (SOAEs) (3, 4). Subjective tinnitus is characterized by an abnormal spontaneous activity within the auditory periphery or the central auditory pathway in the absence of any acoustic stimulation, which is interpreted by the brain as sound (5, 6). A psychophysical characterization of tinnitus serves to quantify loudness and pitch/timbre, which can, in the second step, contribute to the understanding of the underlying pathology (7). For the sake of simplicity, in the following, the term pitch will be applied to pure tones and noises, although the term pitch refers to pure tones only and the equivalent for noises would be timbre. Pitch and loudness of a tinnitus sensation are typically characterized by matching a synthesized sound to the tinnitus percept [see Ref. (8, 9) for an overview]. Pitch matches of tinnitus patients are usually in the rather high-frequency region above 3 kHz and only rarely below 1 kHz (10).

However, patients with Ménière’s disease (MD) seem to be an exception regarding tinnitus pitch. MD is classically characterized by a triad of symptoms: fluctuating hearing loss, episodes of vertigo, and tinnitus (11). In early stages of the disease, tinnitus seems to be related to episodes of vertigo, being more intense before and during attacks. With progression of the disease, which is typically associated with increasing hearing loss and ceasing of vertigo, tinnitus stays and its intensity increases (12–14). Descriptions of tinnitus in MD patients range from roaring over buzzing to ringing (13, 15); except for few MD patients presumably in later stages of the disease, who describe their tinnitus as tonal and containing high-pitched components (12, 13). Overall, studies concluded that tinnitus in the majority of MD patients was confined to lower-frequency ranges below 1 kHz and in particular between 125 and 250 Hz (16–21).

An excess of endolymph volume, termed endolymphatic hydrops, is often suggested to cause MD and specifically symptoms such as tinnitus. A transient endolymphatic hydrops was experimentally induced in rodents by an intense low-frequency (LF) sound (22).

In human participants, transient endolymphatic hydrops induced by an intense LF sound has not been shown directly. However, a post-LF-exposure phenomenon called “Bounce Phenomenon” (BP), lasting only a few minutes, was found in humans. The BP includes a transient tinnitus percept, fluctuating hearing thresholds with transient improvement and subsequent worsening (23–26), and biphasic amplitude level changes in otoacoustic emissions (OAEs) (27–31). As OAEs are sounds emitted by the cochlea due to active amplification processes by outer hair cells (OHCs) (32), the origin of the BP is thought to be cochlear (33). Increased endocochlear potentials in the cochlea have been recorded after presentation of LF sound (22) and were suggested to lead to an increase in the spontaneous firing rate of auditory nerve fibers, resulting in a “rate tinnitus” (34), presumably in the absence of any structural cochlear impairment.

In BP studies, human participants reported a transient “roaring” tinnitus immediately after LF sound exposure (23, 26, 27). The time course and relative loudness of the transient tinnitus percept were characterized with psychophysical studies (26, 30), but tinnitus pitch has not been systematically described with psychophysical measures yet. This study employed a tinnitus-matching procedure to quantify and compare tinnitus percepts in MD patients and normal-hearing (NH) participants after exposure to intense LF sound. Both study groups presumably presented with impaired cochlear ion balance that might manifest in endolymphatic hydrops in MD patients and shows indirectly as BP after LF sound in NH participants.

## Materials and Methods

### Subjects

The study population consisted of two groups: a group of NH participants and a group of patients with MD. In the following, members of both groups will be referred to as participants. NH participants comprised 18 females and 10 males (age range 20–29, mean age 23.7) with no reported hearing problems, no ear surgery, no recent ear infections, and no tinnitus. All NH participants had hearing thresholds of less than 25 dB HL between 0.25 and 8 kHz, tested with a Matlab-based automated procedure [Automatic Pure Tone Audiometry APTA-HF 2012 V2.28 (HörTech, Oldenburg, Germany)]. One ear was pseudo-randomly chosen (14 left ears, 14 right ears) and exposed to a LF sound. The transient tinnitus was lateralized and perceived on the exposed side only.

The group of MD patients consisted of nine females and nine males (age range 26–74, mean age 53.1) diagnosed with definite MD according to the criteria recently formulated by the Bárány Society joint with several national and international organizations (14). Additionally, endolymphatic hydrops had been detected with magnetic resonance imaging after intra-tympanic gadolinium injection (35) before study participation in 15 of 18 patients.

Ménière’s disease patients were included when they were unilaterally affected only (10 left ears, 8 right ears) and when they reported tinnitus localized to the affected ear.

Patients with middle ear disorders, pathologies of the auditory nerve or recent ear infections were excluded from the study. Patients with obvious noise-related hearing damage (i.e., notched audiograms) as well as patients with a history of noise exposure were not eligible either. All MD patients were required to be in a stage of the disease with fluctuating symptoms, with at least one vertigo attack during the 6 months preceding the experiment. In the contralateral, non-affected ear patients were required to have hearing thresholds better than 40 dB HL below 2 kHz, and better than 70 dB HL at higher frequencies.

This study was approved by the ethics committee of the University Hospital of the Ludwig-Maximilians-Universität Munich, Germany, in agreement with the Code of Ethics of the World Medical Association for experiments involving humans (Declaration of Helsinki) and all participants (MD and NH) gave their written informed consent.

Experiments with NH participants were conducted in a double-walled, sound-attenuated booth at the Department of Biology, Ludwig-Maximilians-Universität Munich, Martinsried. Experiments with MD patients were carried out in a sound-attenuated booth at the ENT Department at the University Hospital of the Ludwig-Maximilians-Universität Munich, Germany.

### Signal Generation and Data Acquisition

Signal generation and data acquisition was implemented with scripts written in MATLAB 7.5 (MathWorks, Natick, MA, USA). Sound generation and acquisition was done with an RME Fireface UC 24-bit external sound card (RME, Audio AG, Haimhausen, Germany). The sampling rate was 44.1 kHz. For sound stimulation, SoundMexPro (HörTech GmbH, Oldenburg, Germany) was employed, which enables low-latency multi-channel Audio Stream Input/Output and interactive changes of stimuli properties within the MATLAB environment.

### SOAEs Recording

In NH participants, SOAEs were recorded with the ER-10C distortion product otoacoustic emission (DPOAE) probe microphone (Etymotic Research Inc., Elk Grove Village, IL, USA). The recorded signal was amplified 30 dB by the preamplifier of the external sound card. Level and frequency of SOAEs were recorded in the control trial for 120 s (measured in 21 of 28 NH participants) and after LF stimulation for 240 s (measured in 15 of 28 NH participants). In an artificial ear (B&K 4157, Brüel & Kjaer Sound and Vibration Measurement A/S, Denmark), no artifacts exceeding the noise floor of the system could be detected during recording. A probe-fit-check procedure preceded and concluded each measurement by presenting a band-stop noise consisting of a low- and a high-frequency band and analyzing the ear response using a Fourier transform analysis. If the probe-fit-check procedure at the end of a trial indicated that the probe position had changed, the trial was rejected and repeated.

### Tinnitus-Matching Procedure

In experiments with MD patients, the output of the sound card was sent to HDA 200 headphones (Sennheiser, Wedemark-Wennebostel, Germany). In NH participants, two different sound systems were used for the two ears: an ER4 insert ear phone (Etymotic Research Inc., Elk Grove Village, IL, USA) was used to present the matching stimuli to one ear. An ER-10C DPOAE probe system with an additional tube coupled to an external transducer was used to present LF sounds (30 Hz sine wave, 120 dB SPL, 90 s, including 0.1 s raised-cosine ramps) to the other ear in order to elicit a tinnitus sensation.

The external transducer was a small broadband unit (NSW1-205-8A, Aura Sound Inc., Santa Fe Springs, CA, USA) driven by a RB-960BX power amplifier (Rotel, Worthing, UK). This transducer was connected to a 50-cm long polyethylene tube (inner diameter 1 mm), the tip of which was fed through the foam ear tip of the ER-10C DPOAE probe. The ER-10C includes a microphone for recording sound pressure in the ear canal enabling the examiners to calibrate the LF sound. The amplitude response of the DPOAE probe microphone was compared with the amplitude response in an artificial ear (B&K 4157, Brüel & Kjaer Sound and Vibration Measurement A/S, Denmark) and corrected for deviations. The level of the first harmonic of the LF sound was at least 50 dB lower than the level of the desired LF frequency.

**Figure 1:**
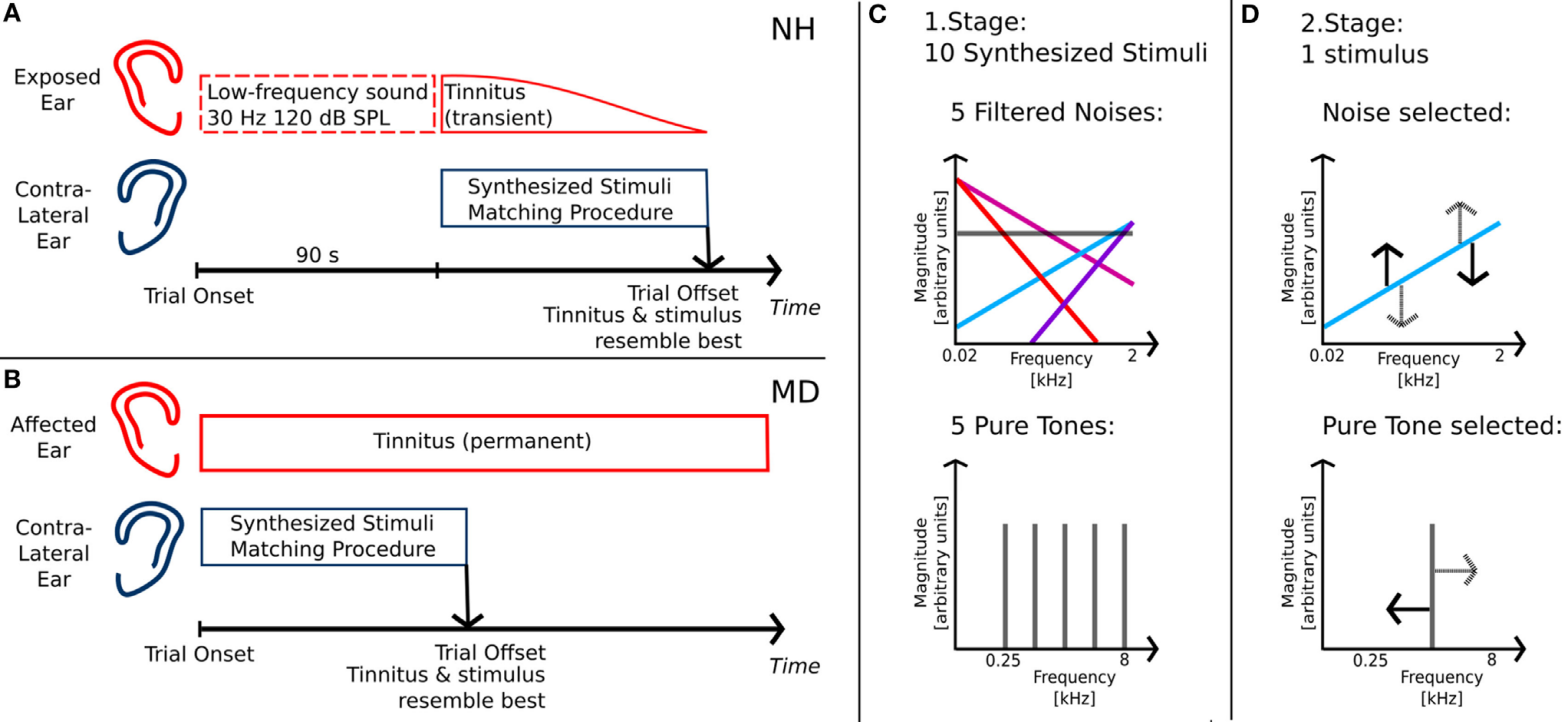
**Tinnitus-matching procedure**. **(A)** Matching procedure for normal-hearing participants with intense low-frequency sound exposure prior to matching; **(B)** matching procedure for Ménière’s disease patients with unilateral disease; **(C)** first stage of matching procedure: determining stimulus class of tinnitus percept; **(D)** second stage of matching procedure: adjusting stimulus regarding pitch/timbre and loudness of tinnitus.

Participants (NH and MD) were given standardized, written and illustrated instructions for the tinnitus-matching procedure. In NH participants, each trial was started with LF-sound exposure to one ear, thereby inducing a transient tinnitus percept in the exposed ear (see Figure 1A). Since the transient tinnitus lasted only for about 90 s after LF exposure (30), NH participants were allowed to restart the LF stimulus playback if necessary. As tinnitus was a unilateral symptom in both groups, matching stimuli were presented to the contralateral, unaffected ear (see Figures 1A,B). Participants were able to interactively adjust matching stimuli regarding loudness and pitch with a gamepad (Bigben Interactive GmbH, Bergheim, Germany). Matching stimuli were continuous and generated in real time by a SoundMexPro plug-in.

The matching procedure was split into two stages. The first stage served to determine whether the participants’ tinnitus was matched best either with a pure tone or with a noise. Therefore, 10 different synthesized stimuli were presented. The stimuli comprised pure tones of five different frequencies, equally spaced on a logarithmic frequency axis between 0.25 and 8 kHz (0.25, 0.595, 1.414, 3.364, and 8.0 kHz) and five noises derived from Gaussian noise with different filter slopes (see Figure 1C). In the following, those slopes are referred to as spectral tilts with units of decibel per octave. They ranged from negative values with dominant LF components (−12 and −6 dB/octave) over white Gaussian noise (0 dB/octave) to positive values with dominant high-frequency components (+6 and +12 dB/octave). Stimuli were filtered such that linear phase and frequency distortions of the transducers (NH participants: ER4, Etymotic Research; MD patients: HDA 200, Sennheiser) were exactly compensated for. Participants started the playback of the individual stimuli successively by choosing from a graphical representation of the stimuli on a user interface. No information regarding the physical properties of the stimuli was available from the graphical representation. The 10 stimuli were identical but reordered on the user interface for each trial.

After having listened to all stimuli, participants were asked to select the stimulus best matching their tinnitus. After stimulus selection, participants had to adjust the stimulus level, such that the selected tone matched their tinnitus as well as possible. Selecting 1 out of the 10 stimuli and adjusting its loudness was counted as one trial in the first stage of the matching procedure. In NH participants, trials were repeated until the same stimulus class was selected in two successive trials. In MD patients, due to time constraints, only two trials were carried out regardless of whether participants selected the same stimulus or different stimuli in those two trials.

In the second stage, participants carried out fine adjustments of level and pitch of either a pure tone or a noise. Matching stimuli were presented with a starting frequency or starting spectral tilt corresponding to the selected stimulus of the first stage, respectively. In MD patients selecting two different stimuli in the first stage, the matching stimulus was chosen based on the patient’s statement of which of the two selected stimuli was the better fit. The starting sound level was the mean sound level derived from the last two trials of the first stage.

During presentation of the matching stimulus, participants could continuously adjust loudness and frequency or spectral tilt (see Figure 1D). When participants found an adjustment for the matching stimulus that resembled their tinnitus, they stopped the adjustment procedure. Subsequently, the adjusted matching stimulus was again presented continuously and participants were required to adjust its sound level such that it was just audible. Similar to a Békésy tracking procedure, participants decreased the level of their adjusted matching stimulus until they did not perceive it anymore and then increased its intensity until they heard the stimulus again. This was done to estimate the sensation level of the matching stimulus. Adjusting the matching stimulus pitch and level and tracking the corresponding hearing threshold was considered as one trial in the second stage of the matching procedure.

In this second matching stage, NH participants always ran five trials and MD patients two to five trials depending on how many they were capable of doing due to time constraints and cognitive load.

After each trial, NH participants and MD patients were asked to describe their transient tinnitus in their own words. In NH participants, measurements were taken on two to four different days and lasted between 25 and 60 min each. In MD patients, testing was embedded in their clinical routine and carried out on 1 or 2 days. Measurements lasted 40–60 min.

All data analysis and statistics were carried out with scripts written in MATLAB 7.5 (MathWorks, Natick, MA, USA). Visualizations were done either with MATLAB 7.5 or Inkscape 0.91 (The Inkscape Team, http://www.inkscape.org).

## Results

### Estimates of Level and Pitch of Tinnitus in NH Participants after LF Sound Exposure

All 28 NH participants exposed to the LF sound experienced a transient tinnitus percept and were able to match its pitch and level with a matching stimulus presented to the contralateral ear. Matching stimuli were adjusted after offset of the LF exposure, on average within 68.7 ± 32.5 s (mean ± SD, *N* = 28). This suggests that most NH participants concluded the matching while still hearing the transient tinnitus, which lasts about 90 s on average (30).

**Figure 2:**
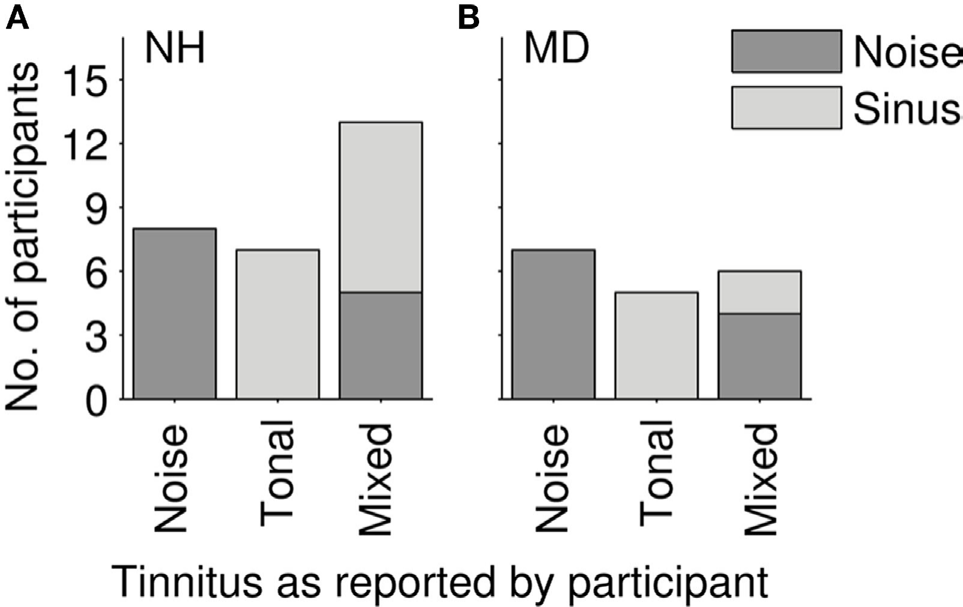
**Distribution of reported tinnitus quality. (A)** Normal-hearing participants (N = 28). **(B)** Ménière`s disease patients (N = 18); gray tones code for the stimulus class that participants selected in the tinnitus-matching procedure (light gray—pure tone; dark gray—noise).

Conclusions drawn from questioning the NH participants after the software-based adjustment procedure are summarized as follows: 7 of the 28 NH participants described their tinnitus as tonal, 8 NH participants as a noise. The remaining 13 NH participants reported a hybrid percept consisting of noise and one or more tones. During the matching procedure, 8 out of those 13 NH participants experiencing a hybrid percept selected a matching tone and only 5 selected a matching noise. Altogether, 15 NH participants selected and adjusted a tone and 13 NH participants selected a noise (Figure 2A). Subjective descriptions of tinnitus perceived by NH participants after LF exposure are summarized in Table 1.

**Table 1:** **Qualitative description of transient tinnitus after intense low-frequency sound exposure (30 Hz, 120 dB SPL, 90 s) by normal-hearing participants (*N* = 28)**.

	*N*
**Noise-like tinnitus**	**8**
*Noise*—exactly like matching noise	5
*Low-Pitched Noise* (Roaring like a fan)	2
*Noise* but tonal character	1
**Tonal tinnitus**	**7**
*Pure Tone*—exactly like matching tone	2
*Two Tones*—alternating/simultaneously (1 tone faint + 1 tone more intense) (Ringing like church bell, Chinese meditation balls)	5
**Hybrid tinnitus percept**	**13**
*Low-Pitched Noise* (Roaring) + tone	5
*High-Frequency Tone*(*s*) + machine-like sound (Rattling, jackhammer, sewing machine)	5
*Other*: noise with low-frequency (LF) modulation	3
LF tone with tinny ring	
Sound of pressure valve	

The loudness of the transient tinnitus percept was reported to be faint up to clearly audible with the tinnitus starting to fade out after a minute. Some participants also described qualitative changes in their tinnitus percept over time. Most of those participants reported a hybrid percept of pure tone(s) and noise. In those cases, the relative contribution of noise and tones to the tinnitus percept shifted over time. The tonal components seemed to fade out over time, while the noise serving as a background noise at the beginning of the BP got more prominent at later time points. Participants did not complain about the percept and its loudness. At most participants stated the tinnitus to be irritating, especially in combination with a feeling of aural fullness.

### Tonal Tinnitus Percepts in NH Participants after LF Exposure

**Figure 3:**
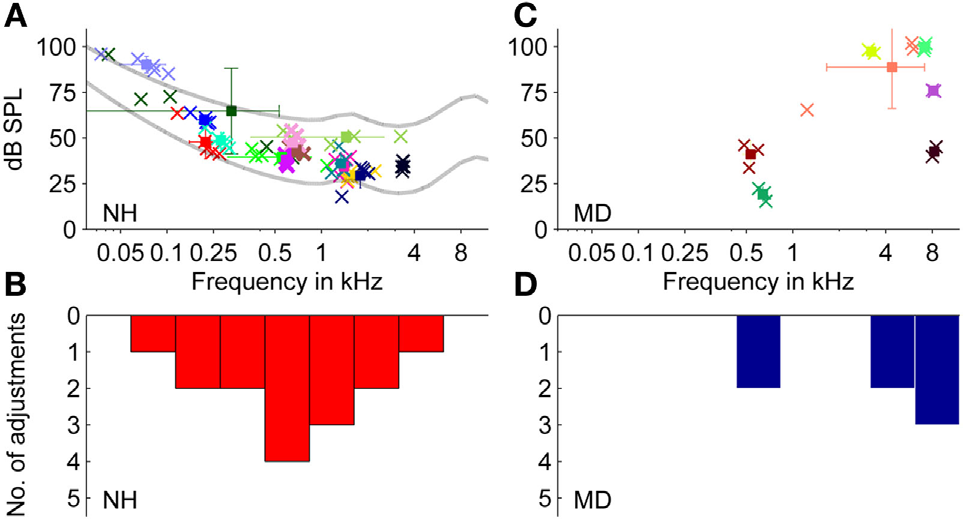
**Adjustments of level and frequency of externally presented pure tones to match a tonal tinnitus percept**. **(A,B)** Normal-hearing participants (*N* = 15). **(C,D)** Ménière’s disease patients (*N* = 7). **(A,C)** Plots show adjusted pure tones of individual participants. Data from individual participants are represented by different colors. Square symbols represent the mean of the two to five individual adjustments of one participant (indicated by cross symbols). Error bars represent the SD regarding sound pressure level (dB SPL) (vertically) and frequency (Hz) (horizontally). Lines in **(A)** represent equal-loudness contours for loudness levels at 25 and 60 phons. **(B,D)** Distribution of the selected matching tone frequencies of data shown in **(A,C)**, respectively (bin width = 1,249 cents).

Fifteen NH participants selected pure tones to characterize their tonal tinnitus. The pure tone frequency was determined by averaging all adjustments per subject. The selected matching tone frequencies (see Figures 3A,B) show an accumulation between 0.1 and 2 kHz, where 14 out of 15 participants selected matching tones below 2 kHz.

The mean frequency of the 15 averaged pure tones chosen was 0.96 ± 0.89 kHz (mean ± SD, *N* = 15). As the SD across adjustments in hertz is only of limited value, frequency deviations were also expressed logarithmically as fraction of an octave in cent, where 100 cents equal a semitone, so that 1,200 cents correspond to an octave.

In 13 of 15 NH participants, SDs for single participants comprised less than 1,200 cents, and for some participants, the SD was as low as 30 cents. The inter-subject mean of the sound pressure level of the adjusted matching tone was around 49.5 ± 15.9 dB SPL (mean ± SD, *N* = 15).

**Figure 4:**
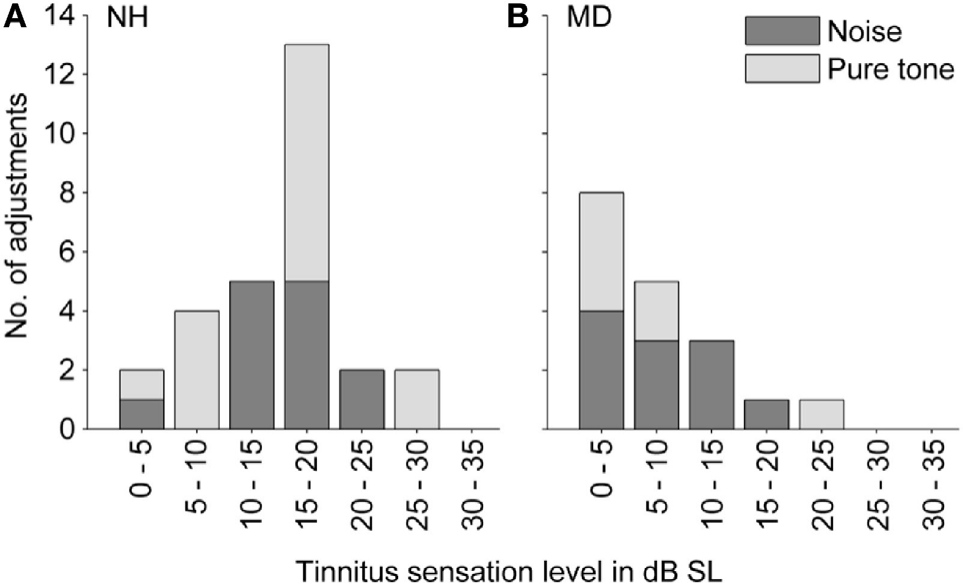
**Distribution of tinnitus loudness (expressed as sensation level). (A)** Normal-hearing participants (N = 28). **(B)** Ménière’s disease patients (N = 18). Colors code for the stimulus class that participants selected in the tinnitus-matching procedure (light gray – pure tone; dark gray – noise).

Sound levels for matching tones decreased with increasing frequency from 90 dB SPL at 70 Hz to 30–35 dB SPL at 2–4 kHz. Matched pure tone levels followed equal-loudness contours for human hearing within a loudness level range of 25–60 phons (see Figure 3A). Thus, for NH participants, matching tones at lower frequencies were not generally perceived louder than at higher frequencies, despite the difference in sound pressure levels. For an interpretation of how loud participants (NH and MD) perceived their tinnitus, sensation levels were calculated (see Figure 4A). The sensation level is the difference (in decibel) between the presented sound level and the absolute hearing threshold for the same sound. The mean sensation level for tonal tinnitus matches of NH participants was 15.2 ± 6.7 dB SL (mean ± SD, *N* = 15).

### Noise-Like Tinnitus Percepts in NH Participants after LF Exposure

**Figure 5:**
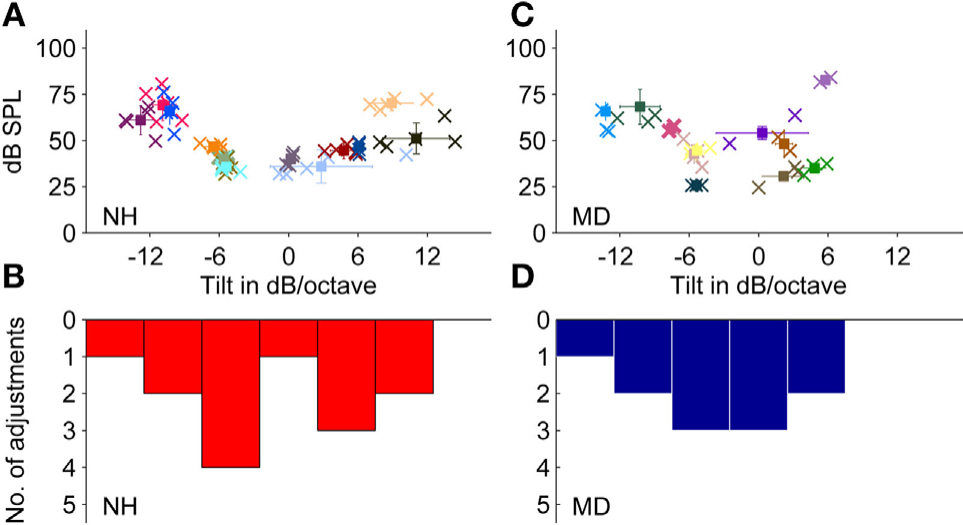
**Adjustments of level and frequency content of externally presented noises to match a noise-like tinnitus percept**. **(A,B)** Normal-hearing participants (*N* = 13). **(C,D)** Ménière’s disease patients (*N* = 11). **(A,C)** Plots show adjusted noises of individual participants. Error bars represent the SD regarding sound pressure level (dB SPL) (vertically) and frequency content (dB/octave) (horizontally). **(B,D)** Distribution of the matching noise tilts of the data shown in **(A,C)**, respectively, within the range of −17.5 to +17.5 dB/octave (bin width = 5 dB/octave). Color coding and symbols as in Figure 3.

Thirteen NH participants chose noises to match their LF-induced transient tinnitus. Adjusted spectral tilts showed a bimodal distribution slightly shifted toward negative tilts with an average adjusted spectral tilt of −1.8 ± 7.9 dB/octave (mean ± SD, *N* = 13) (see Figures 5A,B). Except for one participant choosing white noise (mean tilt: ~0 dB/octave) to match the tinnitus percept, all NH participants selected either noises with a clearly positive tilt (mean ± SD = 6.7 ± 3.3 dB/octave, *n* = 5) or with a clearly negative tilt (mean ± SD = −8.1 ± 3.1 dB/octave, *n* = 7). Within-participant variability between adjustments was quite small (SD between 0.02 and 4.4 dB/octave).

Sound levels of the matching noises were within the range of 36–70 dB SPL with a mean of around 49.5 ± 12.9 dB SPL (mean ± SD, *N* = 13). Matching noises with highest sound pressure levels were noises with the steepest spectral tilts, either negative or positive. The mean sensation levels of matching noises were 15.3 ± 5.6 dB SL (mean ± SD, *N* = 13).

Sensation levels of both selected matching tones and matching noises in NH participants were summarized (see Figure 4A), and the mean was calculated as 15.2 ± 6.1 dB SL (mean ± SD, *N* = 28).

### Estimates of Level and Pitch of Tinnitus in MD Patients

In all 18 MD patients, the tinnitus was a permanent sensation, sometimes varying in loudness over time. In eight patients, the loudness of the tinnitus was apparently correlated to the vertigo attacks increasing right before and/or during an attack. Three patients reported not only a loudness change correlated to the attacks but also an increase of tinnitus pitch. In this case, patients were asked to match their current tinnitus percept. Seven patients reported their tinnitus to be a noise, and five patients reported it to be a pure tone. The other six patients reported to hear a hybrid percept of pure tone(s) and noise. In the tinnitus-matching procedure, two of those patients perceiving a hybrid tinnitus matched their tinnitus with a pure tone and four of them with a noise (see Figure 2B). Qualitative tinnitus descriptions by the patients are summarized in Table 2. Descriptions included low-pitched noise, modulated noise, and high-frequency whistling.

**Table 2:** **Qualitative description of tinnitus by Ménière’s disease patients (*N* = 18) with unilateral disease and one-sided, permanent tinnitus**.

	*N*
**Noise-like tinnitus**	**7**
*Low-pitched nois*e (roaring, buzzing)	4
*Noise with modulation/pulsatile* (rushing blood)	2
*Other: humming*	1
**Tonal tinnitus**	**5**
*Pure Tone*—exactly like matching tone	2
*High-frequency whistling, swishing*	3
**Hybrid tinnitus percept**	**6**
*High-frequency tone*(*s*) + *low-pitched noise* (angle sander, whistling, and dull roaring)	4
*Other*: rushing blood	2
Swarm of bees	

### Tonal Tinnitus in MD Patients

Seven MD patients selected pure tones in the matching procedure. Thereby, data did not show the previously suggested correlation that older patients or patients in later stages of the disease were more likely to experience tonal tinnitus of high-frequency quality instead of noise-like tinnitus (linear correlation coefficients, age: *r* = −0.12, *p*-value = 0.64, stage of disease *r* = 0.08, *p*-value = 0.76).

Selected matching tone frequencies showed a bimodal distribution with peaks around 500 Hz and around 5 kHz (see Figures 3C,D). The mean frequency was 4.6 ± 3.3 kHz (mean ± SD, *N* = 13).

Matched sound levels increased with increasing frequency from 20 dB SPL at 0.6 kHz to 80–100 dB SPL at 7–8 kHz. Sensation levels of matching tones, however, were small (mean ± SD = 7.0 ± 7.8 dB SL, *N* = 7).

### Noise-Like Tinnitus Percepts in MD Patients

Eleven of 18 MD patients selected noises to describe their tinnitus percept. The matching noise properties were averaged across the two to three trials for each patient.

Across patients, the average spectral tilt of matched noises was −2.9 ± 6.3 dB/octave (mean ± SD, *N* = 11). Matching noises showed adjusted tilts between −13 and +4 dB/octave and resulted in an almost normal distribution with a shift toward negative tilts (see Figures 5C,D).

On average, the noise level adjusted by MD patients was 50.4 ± 17.3 dB SPL (mean ± SD, *N* = 11), which was comparable to noise levels of noises matched by NH participants with transient tinnitus. Due to hearing loss in the majority of MD patients, the sensation levels of the matching tones were with 8.5 ± 4.9 dB SL (mean ± SD, *N* = 11) significantly lower than in NH participants (Mann–Whitney test, *p*-value = 0.0002).

Sensation levels of both matched tones and matched noises in MD patients were summarized (see Figure 4B), and the mean was 7.9 ± 6.0 dB SL (mean ± SD, *N* = 18).

### Comparison of Tinnitus Percepts in MD Patients and in NH Participants after LF-Sound Exposure

#### Tonal Tinnitus Percepts

Pure tones matched by MD patients and matched by NH participants differed in their distribution (see Figure 3) (two-sample Kolmogorov–Smirnov test, *p*-value = 0.019). While the majority of MD patients chose pure tones above 3 kHz, which is comparable to most tinnitus percepts of tinnitus patients (10, 16), the majority of NH participants selected pure tones below 2 kHz.

#### Noise-Like Tinnitus Percepts

Comparing the two subgroups of MD patients selecting noises and NH participants with BP-induced tinnitus selecting noises did not show pronounced differences. Although the distribution appeared bimodal for NH participants and rather normally distributed in MD patients, the null hypothesis stating that the two subgroups were from equal distributions could not be rejected (two-sample Kolmogorov–Smirnov test; *p*-value = 0.86). Both subgroups had a tendency to select noises with a negative spectral tilt, which corresponds to previous reports from the literature (16–18, 23, 30).

### Test–Retest Reliability

In general, the pitch and loudness matches obtained with the current two-stage procedure showed good test–retest reliability. Intra-class correlation coefficients (ICC) were calculated for the five trials of fine matching run in one to three different sessions in NH participants (pitch matching: ICC = 0.92; loudness matching: ICC = 0.85). In MD patients, data were usually collected in one session only, but for the two to three different adjustments ICC indicated very strong test–retest reliability, too (pitch matching: ICC = 0.93; loudness matching: ICC = 0.92).

### SOAE Measurements in NH Participants

**Figure 6:**
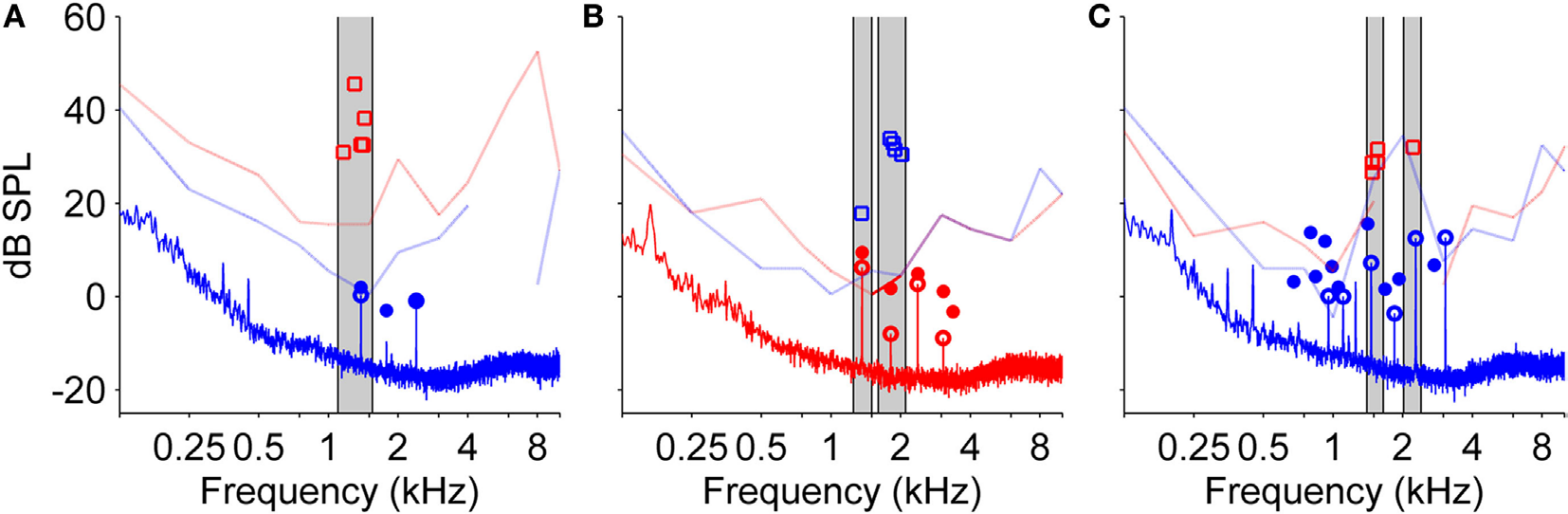
**Audiograms from both ears, spontaneous otoacoustic emissions (SOAEs), and level and frequency estimates of the tinnitus percept after low-frequency (LF) sound exposure for three different normal-hearing participants (A–C)**. SOAEs were recorded in ipsilateral ears before and immediately after exposure to a 30 Hz tone at 120 dB SPL for 90 s. In all three measures, red color refers to the right ear, blue color refers to the left ear. Light lines indicate audiogram thresholds (gaps within light lines are due to lack of valid data points of audiometric testing), open squares represent five adjustments of level and frequency of the matching tone presented to the contralateral ear. Solid lines represent the spectrum of the SOAE recording. Open circles represent SOAEs before LF sound exposure, filled circles represent SOAEs after LF sound exposure. Gray patches highlight SOAEs and corresponding matching tone adjustments.

In 15 of the 28 NH participants, SOAEs were measured in the same ear that was exposed to the LF sound. The first measurement was run before LF exposure to find SOAEs. The second SOAE measurement was carried out immediately after LF exposure during the BP, while NH participants perceived the corresponding transient tinnitus. During the BP, “new” SOAEs could develop and existing SOAEs increased in level. Occasionally, those SOAEs exceeded the hearing threshold determined for the same ear by audiometric testing at the beginning of the experiments. In 10 NH participants, SOAEs could be found. In all of those participants, SOAEs were slightly altered when recorded during the BP compared to baseline before LF exposure. In eight NH participants, SOAE levels exceeded the individual hearing threshold at the SOAE characteristic frequency during the BP. From these eight participants, five matched their tinnitus with a pure tone, three with a noise. Figure 6 shows SOAEs, hearing threshold and selected tinnitus-matching tones from three participants with “audible” SOAEs. These data suggest that some NH participants might possibly have matched their tinnitus to a transiently audible SOAE.

## Discussion

The current study, by applying a comprehensive tinnitus-matching procedure, compared tinnitus in two groups with chronic (MD patients) and transiently (NH participants after LF exposure) challenged cochlear homeostasis presumably resulting in hydropic conditions.

Our results show that sensation levels of tinnitus percepts were on average between 8 dB SL (MD patients) and 15 dB SL (NH participants after LF sound). Tinnitus pitch varied substantially both within and across MD patients and NH participants. While MD patients matched their tinnitus mainly with either high-pitched pure tones or noises with a tendency to low-pitched (roaring) noises, NH participants perceiving a transient tinnitus after LF-exposure matched that tinnitus with high- or low-pitched noises or low-pitched pure tones. In the few studies available in the literature, MD patients were found to perceive mainly LF tinnitus (16–21), albeit reports on MD patients with high-frequency tinnitus exist (12, 13, 36). Tinnitus percepts of MD patients in the current study were more diverse, and this is probably due to the following reasons: first, the matching procedures employed in the available literature differed from our advanced matching paradigm, in that participants could adjust matching stimuli on their own without any interference or bias caused by the examiner. Furthermore, participants could choose between two matching stimulus classes (pure tones and noises). Second, participating MD patients were heterogeneous regarding age (26–74 years). As the onset of MD symptoms generally occurs around 30–50 years (37, 38), younger MD patients without age-related hearing loss are underrepresented in our patient population. Nevertheless, contralateral, unaffected ears of all participating patients were within the normal hearing range when considering the age-corrected pure-tone average at 2, 3, and 4 kHz. This is important as the matching stimulus was delivered to the unaffected ear. All patients, despite their age, were in a stage of the disease with fluctuating symptoms.

In the following, we will dissect tinnitus percepts into tonal and noise-like tinnitus and discuss tinnitus loudness for both kinds of percepts in the end. Underlying mechanisms possibly generating the tinnitus percepts in both MD patients and NH participants after LF exposure will be proposed for tonal and noise-like tinnitus separately.

### Tonal Tinnitus Generation

MD patients mainly chose high-frequency matching tones when their tinnitus was predominantly tonal. This is comparable to the tinnitus quality typically related to inner hair cell (IHC) and OHC damage (39). Here, it was suggested that the tinnitus frequency roughly corresponds to characteristic frequency of the impaired hair cells or to the frequency at the boundary between intact and impaired hair cells (40, 41). This tinnitus mechanism might generate the tonal tinnitus percepts MD patients perceive. Audiograms of MD patients often show LF hearing loss but high-frequency hearing loss also occurs, mostly in later stages of the disease (12, 13). The majority of MD patients in this study showed greatly reduced DPOAE levels as judged from measurements acquired during the clinical routine. This indicates some degree of OHC function impairment, but it is unclear if this is age-related or due to the presence of endolymphatic hydrops. OHC loss might not suffice to induce tinnitus. Therefore, damage to IHCs or the auditory nerve, which cannot be detected with DPOAE measurements, might also be required. Given the presence of both IHC and OHC dysfunction, tonal tinnitus could also be the result of endolymphatic hydrops (or its underlying pathology) combined with the pre-existing hair cell dysfunction.

Tonal tinnitus in NH participants after LF exposure showing frequencies below 2 kHz is presumably triggered by transiently challenged cochlear homeostasis. Intense, LF sound causes broad excitation patterns peaking at the apical part of the cochlea. Thus, effects on cochlear homeostasis are not restricted to the characteristic frequency region of LF sound and higher-frequency regions can be affected as well.

Temporary tinnitus in humans is generally thought to be caused by temporary decrease of OHC amplification, which results in increased firing in the central auditory system and tinnitus generation (42). Although this generation mechanism cannot be ruled out, especially for participants showing temporary worsening of hearing threshold after LF exposure, it seems to be unlikely because of concurrent, increasing OAE levels typically following LF exposure due to enhanced OHC amplification (30). Temporary enhanced amplification and increased OAE levels might contribute to a different generation mechanism.

In three participants, it could be shown that frequencies of the selected matching tones roughly corresponded to the recorded SOAE frequencies. Besides the fact that permanent tinnitus percepts originating from an SOAE are rare [between 2 and 4.5% (4, 43)], SOAEs seem to be a plausible explanation in the case of transient tonal tinnitus after LF sound exposure for the following reasons:

Tinnitus frequencies selected by NH participants in the present study correspond to typical SOAE frequencies. SOAEs in human adults are usually in the range between 0.9 and 4 kHz with distribution maxima near 1.5 and 3 kHz (44, 45).

Under normal conditions, SOAEs are relatively stable in both frequency and level (46). After intense LF exposure, however, frequency and level of SOAEs slowly cycle for a time period of about 2 min in a stereotypic manner with a level maximum typically occurring about 50 s after LF sound offset (27, 31). SOAEs presumably buried in the noise floor become detectable during that period. Typically, SOAEs are not perceived by their owner (47). It has been shown, however, that induced frequency or level changes of SOAEs can result in SOAEs becoming transiently audible (47–49), as the inhibition of SOAE perception at higher stages of auditory processing (45) might not be active anymore. Not all NH participants perceiving tonal tinnitus had SOAEs with frequencies similar to the chosen matching tone frequency. NH participants with recordable SOAEs often showed more than one SOAE, which would have resulted in a percept difficult to match with a single pure tone.

In NH participants without recordable SOAEs, SOAE levels might be significantly underestimated by external recordings after OAE back-propagation, compared to sound pressure level of OAEs within the cochlea (47). This is strongly supported by the comparison of DPOAE sound levels measured in the gerbil meatus and the electrophysiological response to the same DPOAE in the gerbil cochlear nucleus (50). Thus, for SOAEs to be perceived by their owner, it might not be necessary that SOAE levels, as measured in the meatus, exceed the individual hearing thresholds at the SOAE characteristic frequency.

### Noise-Like Tinnitus Generation

In both subject groups, a major proportion of tinnitus matches indicated a noise-like tinnitus. In experimental animals, it has been shown that the endocochlear potential increases temporarily with intense LF exposure (22, 51), resulting in a depolarization of IHCs and consequently in an increased spontaneous activity of the auditory nerve (34).

While direct recordings of the endolymphatic potential in MD patients are not feasible, a similar mechanism is nonetheless conceivable. Increased spontaneous activity of the auditory nerve could lead to the perception of a tinnitus, for which Patuzzi (34) coined the term “rate tinnitus.” If a large expanse of the cochlea was affected by the above mechanism, a noise-like percept would result.

MD patients perceiving noise-like tinnitus percepts chose mainly noises with negative tilts (low pitches). This is in line with reports that MD patients typically show LF hearing loss at frequencies below 3 kHz (14), suggesting that, for hitherto unknown reasons, the apical part of the cochlea is most affected, resulting in a LF rate tinnitus. But, presumably depending on the duration of the disease, audiograms of MD patients reveal a broad range of affected frequencies (15), which is also reflected in our study population. This might explain why MD patients selected matching noises not necessarily limited to the LF range.

During intense LF stimulation, cochlear excitation is not restricted to the characteristic frequency, but the whole cochlea is excited and individual differences in cochlear excitation patterns might exist. LF stimulation affects the apical end of the cochlea strongest and LF components should be more prominent in noise-like tinnitus after intense LF exposure in NH participants (corresponding to selected noises with negative tilt). Those NH participants that chose high-pitched noises to match their LF-induced tinnitus may have done so as a compromise to represent hybrid tinnitus percepts consisting of both noise and tonal components or cochlear locations other than the apex dominate the tinnitus percept.

### Tinnitus Sensation Levels

Sensation levels of tinnitus percepts were estimated with previously selected matching stimuli. In MD patients, hearing thresholds even on the contralateral, unaffected ear were slightly elevated at higher frequencies due to age-related hearing loss. Therefore, for MD patients choosing a high-pitched pure tone to match their tinnitus, loudness matches might have resulted in lower values than loudness matches using frequencies at which hearing was normal (8, 52). Loudness recruitment could also contribute: for participants suffering from cochlear hearing loss, low to moderate sensation levels may be much louder than for NH participants because the lack of cochlear compression decreases the overall dynamic range of loudness perception dramatically (52, 53). Furthermore, low sensation levels of matching tones in MD patients can be due to tinnitus loudness fluctuations. Patients were measured between vertigo attacks when tinnitus loudness was typically lower than immediately before or during attacks.

On the other hand, high loudness values in NH participants could be explained with a shift of attention toward the suddenly occurring tinnitus percept. Tinnitus sensation levels after LF exposure were on average higher than tinnitus sensation levels in MD patients and higher than tinnitus sensation levels in most patients with pathologies other than MD. Studies showed that tinnitus loudness was generally matched with stimuli below 10 dB SL, even in participants referring to their tinnitus as loud (8, 10, 54).

### Procedural Limitations

Participants were presented with a limited range of sounds from which they had to select. Although this limited range of sounds was chosen to facilitate the matching procedure, participants complained about not being able to adjust amplitude modulations or about being restricted to one stimulus class when hearing hybrid percepts with both pure tones and noises. Furthermore, for both MD patients and NH participants with an LF-induced transient tinnitus, tinnitus loudness, and sometimes even tinnitus pitch were varying over time. In case of MD patients, these variations can take place over days or weeks depending on vertigo attacks or the current stage of the disease (12, 13). In NH participants, fluctuations appear within the 1–2 min of tinnitus duration. This is inherent to the transient percept after LF exposure. To guarantee consistency, NH participants were able to retrigger the tinnitus percept by turning on the LF stimulation again.

In either case, tinnitus-matching results can only represent approximations of actual tinnitus percepts, constrained by both choice of offered stimuli and experimental procedures.

## Conclusion

Estimates of tinnitus pitch reported in the literature are heterogeneous and depend heavily on the methods used. Here, we implemented a tinnitus-matching procedure with fewer constraints than in previous, comparable studies, including noises with adjustable spectral shapes. As a consequence, our results revealed a relatively large proportion of participants with noise-like tinnitus, which might have not been detected in previous studies mostly employing sinusoidal matching tones. Contrary to reports in the literature, tinnitus pitch in NH participants after LF sound exposure and in MD patients is not exclusively LF, and hybrid percepts with noise-like and tonal components can occur.

Noise-like tinnitus cannot easily be explained with localized damage to cochlear regions. Rather, a mechanism affecting a broad frequency range is needed. An increase of spontaneous activity of the auditory nerve was suggested to cause the noise-like tinnitus observed here. However, further experiments are now required to identify unusual patterns in auditory nerve spontaneous activity as a potential tinnitus generator in chronic and induced hydropic conditions.

## Author Contributions

MU contributed to study design, performed data acquisition, statistical analysis and interpretation of results, drafting of the manuscript, revised the manuscript, and approved the final manuscript. LW contributed to study design and data acquisition, critically reviewed and approved the final manuscript. EK contributed to data acquisition, revised and approved the final manuscript. RG contributed to data acquisition, revised and approved the final manuscript. MD conceptualized and designed the study, contributed to data acquisition, interpretation of results, drafting of the manuscript, critically reviewed and approved the final manuscript. All authors are agreeable to be accountable for the content of the work, integrity, and accuracy of the data.

## Conflict of Interest Statement

The authors declare that the research was conducted in the absence of any commercial or financial relationships that could be construed as a potential conflict of interest.

## Abbreviations

BP, bounce phenomenon; DPOAE, distortion product otoacoustic emission; ICC, intra-class correlation coefficients; IHCs, inner hair cells; LF, low-frequency; MD, Ménière’s disease; NH, normal-hearing; OAE, otoacoustic emission; OHCs, outer hair cells; SOAE, spontaneous otoacoustic emission.
